# Supplementary material for: Frailty in randomized controlled trials of glucose-lowering therapies for type 2 diabetes: An individual participant data meta-analysis of frailty prevalence, treatment efficacy, and adverse events
Source: PLoS Med. 2025 Apr 7;22(4):e1004553. doi: 10.1371/journal.pmed.1004553 (PMC12052138; doi:10.1371/journal.pmed.1004553)
Supplement: S1 Appendix — (DOCX) [file pmed.1004553.s001.docx]

Supplementary appendix – detail on review process, study selection and analysis pre-specification

# Protocol links

The original protocol for the systematic review within which this analysis is nested including detailed inclusion/exclusion criteria are provided here: doi:10.1136/bmjopen-2022-066491

Full details of the screening and selection of eligible studies are provided here in the first results paper from this project which details the results of screening, quality assessment, and all baseline characteristics of included studies: https://doi.org/10.1101/2024.06.23.24309242 . Full trial level details of all included studies (including each of those included in this analysis of the frailty index) are publicly available here: https://github.com/Type2DiabetesSystematicReview/nma_agesex_public

The assessment of frailty within these studies was a sub-study of the overall systematic review. This sub-study was prespecified and conducted according to its own protocol. Full details of the protocol for the identification of frailty within these trials is provided here: https://zenodo.org/doi/10.5281/zenodo.12528406

The process for identifying and deciding on each of the included deficits within the analysis is described at the link above along with pre-specification of all definitions used. The definition of each of the deficits that were included in the final analysis are detailed below.

# Definition of deficits

Cardiometabolic comorbidities

| Chronic kidney disease | Present = 1, absent = 0 |
| --- | --- |
| Dyslipidaemia | Present = 1, absent = 0 |
| Heart failure | Present = 1, absent = 0 |
| Hypertension | Present = 1, absent = 0 |
| Ischaemic heart disease | Present = 1, absent = 0 |
| Peripheral vascular disease | Present = 1, absent = 0 |
| Stroke | Present = 1, absent = 0 |
| Venous thromboembolism | Present = 1, absent = 0 |
| Valvular heart disease | Present = 1, absent = 0 |

Non-cardiometabolic comorbidities

| Cancer | Present = 1, absent = 0 |
| --- | --- |
| Chronic obstructive pulmonary disease | Present = 1, absent = 0 |
| Dementia | Present = 1, absent = 0 |
| Depression | Present = 1, absent = 0 |
| Diverticular disease | Present = 1, absent = 0 |
| Epilepsy | Present = 1, absent = 0 |
| Glaucoma | Present = 1, absent = 0 |
| Gout | Present = 1, absent = 0 |
| Hepatic disorders | Present = 1, absent = 0 |
| Inflammatory arthritis | Present = 1, absent = 0 |
| Osteoarthritis | Present = 1, absent = 0 |
| Osteoporosis | Present = 1, absent = 0 |
| Parkinsonism | Present = 1, absent = 0 |
| Peripheral neuropathy | Present = 1, absent = 0 |
| Thyroid disorder | Present = 1, absent = 0 |

Laboratory deficits

| Haemoglobin (g/L) | Men: <130 = 1,  >175 = 1  Women: <120 = 1,  >160 = 1 |
| --- | --- |
| Platelets (× 10^9^/L) | <150 = 1,  >400 = 1 |
| Neutrophils (× 10^9^/L) | <1.5 = 1,  1.5-8.0 = 0,  >8.0 = 1 |
| Estimated glomerular filtration rate (mL/min/1.73m^2^) | <30 = 1,  <60 = 0.5 |
| Sodium (mmol/L) | <135 = 1,  135-145 = 0,  >145 = 1 |
| Potassium (mmol/L) | <3.5 = 1,  3.5-5.0 = 0,  >5 = 1 |
| Calcium (mmol/L) | <2.1 = 1,  2.1-2.6 = 0,  >2.6 = 1 |
| Uric acid (mg/dl) | <=7 = 0,  >7 = 1 |
| C-reactive protein (mg/L) | <=5 = 0,  >5 = 1 |
| Albumin (g/L) | <34 = 1,  34-55 = 0,  >55 = 1 |
| Bilirubin (micromol/L) | <=21 = 0,  >21 = 1 |
| AST/ALT (U/L) | AST >33 or ALT >36 = 1,  otherwise 0 |
| Alkaline phosphatase (IU/L) | <=147 = 0,  >147 = 1 |
| LDL cholesterol (mmol/L) | <=3 = 0,  >3 = 1 |
| Uric acid | <300 = 0,  >=300 = 1 |

Functional measures/symptoms

| Self-rated health (scored 0-100) | Scaled to between 0 and 1 |
| --- | --- |
| Mobility | No problem = 0, slight problem = 0.25, moderate problems = 0.5, severe problems = 0.75, unable = 1 |
| Self-care (washing/dressing) | No problem = 0, slight problem = 0.25, moderate problems = 0.5, severe problems = 0.75, unable = 1 |
| Usual activities | No problem = 0, slight problem = 0.25, moderate problems = 0.5, severe problems = 0.75, unable = 1 |
| Pain/discomfort | None = 0, slight = 0.25, moderate = 0.5, severe = 0.75, extreme = 1 |
| Anxiety/depression | None = 0, slight = 0.25, moderate = 0.5, severe = 0.75, extreme = 1 |
| Difficulty picking up objects (IWQOL) | Never = 0, rarely = 0.25, sometimes = 0.5, frequently = 0.75, always = 1 |
| Difficulty tying shoes (IWQOL) | Never = 0, rarely = 0.25, sometimes = 0.5, frequently = 0.75, always = 1 |
| Difficulty getting up from chairs (IWQOL) | Never = 0, rarely = 0.25, sometimes = 0.5, frequently = 0.75, always = 1 |
| Difficulty using stairs (IWQOL) | Never = 0, rarely = 0.25, sometimes = 0.5, frequently = 0.75, always = 1 |
| Difficulty dressing (IWQOL) | Never = 0, rarely = 0.25, sometimes = 0.5, frequently = 0.75, always = 1 |
| Feel short of breath (IWQOL) | Never = 0, rarely = 0.25, sometimes = 0.5, frequently = 0.75, always = 1 |
| Painful stiff joints (IWQOL) | Never = 0, rarely = 0.25, sometimes = 0.5, frequently = 0.75, always = 1 |
| Swollen ankles/legs (IWQOL) | Never = 0, rarely = 0.25, sometimes = 0.5, frequently = 0.75, always = 1 |
