# Supplementary material for: Frailty in randomized controlled trials of glucose-lowering therapies for type 2 diabetes: An individual participant data meta-analysis of frailty prevalence, treatment efficacy, and adverse events
Source: PLoS Med. 2025 Apr 7;22(4):e1004553. doi: 10.1371/journal.pmed.1004553 (PMC12052138; doi:10.1371/journal.pmed.1004553)
Supplement: S2 Appendix — (DOCX) [file pmed.1004553.s002.docx]

# Analysis code

# load required packages

library(tidyverse)

library(readxl)

library(multinma)

library(flexsurv)

library(ggridges)

library("viridis")

library(rms)

library(meta)

library(forestplot)

# Distribution of frailty index per trial #####

# read in data with parameters of generalised gamma distribution for

# frailty index in each trial

dist <- read_csv("frailty_index_distributions.csv")

# FI generating data for the curve showing the FI distribution

# based on the cumulative density function of the generalised gamma distribution

fi_dist <- function(y){

df <- data.frame(study = dist$nct_id[y],

fi = rgengamma(1000,

mu = dist$mu[y],

sigma = dist$sigma[y],

Q = dist$Q[y]),

mean = mean_gengamma(mu = dist$mu[y],

sigma = dist$sigma[y],

Q = dist$Q[y]),

centile = qgengamma(p = 0.99,

mu = dist$mu[y],

sigma = dist$sigma[y],

Q = dist$Q[y]))

df

}

# generate the data

fig_3_data <- map_df(1:34, fi_dist)

# read in summary demographic data for plot

trial_data <- read_excel("trial details for plot.xlsx")

# generate figure 3

text_for_fig <- trial_data%>%

rename(study = trial)%>%

left_join(test4%>%distinct(study, study2, mean, centile))%>%

mutate(n_ch = as.character(n),

age = as.character(round(mean_age,1)),

mean_fi = as.character(round(mean, 2)),

centile_fi = as.character(round(centile, 2)),

fi = NA)%>%

distinct(study2, mean, n_ch, age, mean_fi, centile_fi, fi)%>%

filter(!is.na(centile_fi))

heading <- data.frame(study2 = " ",

mean = 0.001,

n_ch = "Total n",

age = "Mean age",

mean_fi = "Mean FI",

centile_fi = "99th centile FI")

fig_3_data <- fig_3_data%>%

left_join(trial_data%>%rename(study = trial))%>%

mutate(population2 = case_when(population == "Chronic renal impairment" ~ "Chronic renal impairment",

population == "drug naive, raised HbA1c" ~ "Drug naive",

population == "dual therapy, raised HBA1c" ~ "On oral therapy",

population == "monotherapy, raised HbA1c" ~ "On oral therapy",

population == "Older people, raised HbA1c" ~ "Older people (>70)",

population == "on insulin, raised HbA1c" ~ "On insulin",

population == "Severe chronic renal impairment" ~ "Chronic renal impairment"))%>%

mutate(comparison2 = gsub("Empagliflozin with metformin vs Metformin or Empagliflozin",

"Empagliflozin vs Metformin",

comparison),

comparison2 = gsub("Placebo/Sitigliptin",

"Placebo",

comparison2))%>%

mutate(study2 = paste(study, comparison2))%>%

mutate(population_order = case_when(population2 == "Chronic renal impairment" ~ 5,

population2 == "Drug naive" ~ 1,

population2 == "On oral therapy" ~ 2,

population2 == "On insulin" ~ 3,

population2 == "Older people (>70)" ~ 4))%>%

full_join(text_for_fig)%>%

full_join(heading)

ggplot(fig_3_data, aes(x = fi, y = reorder(study2, -mean), fill = factor(population_order)))+

geom_density_ridges()+

geom_text(aes(x = 0.48, y = reorder(study2, -mean), label = n_ch), vjust = -0.4, hjust = 0)+

geom_text(aes(x = 0.55, y = reorder(study2, -mean), label = age), vjust = -0.4, hjust = 0)+

geom_text(aes(x = 0.62, y = reorder(study2, -mean), label = mean_fi), vjust = -0.4, hjust = 0)+

geom_text(aes(x = 0.69, y = reorder(study2, -mean), label = centile_fi), vjust = -0.4, hjust = 0)+

xlim(0, 0.8)+

scale_fill_viridis(discrete = TRUE, option = "D",

labels = c("Drug naive", "On oral therapy", "On insulin",

"Older people (>70)", "Chronic renal impairment"))+

labs(y = "Trial and comparison", x = "Frailty index", fill = "Target population",

title = "Frailty index distribution")+

theme(panel.grid.major = element_blank(), panel.grid.minor = element_blank(),

panel.background = element_blank(), axis.line = element_line(colour = "black"))

# data for results text #####

# function to apply FI cut points 0.1, 0.2, 0.3, and 0.4

fi_cutoff2 <- function(y){

df <- data.frame(study = dist2$nct_id[y],

above_0.24 = (1-pgengamma(q = 0.24,

mu = dist2$mu[y],

sigma = dist2$sigma[y],

Q = dist2$Q[y]))*100)

df

}

dist2 <- dist%>%

filter(model == "over_65s")

for_text2 <- map_df(1:34, fi_cutoff2)%>%

left_join(test5%>%distinct(study, population2, mean_age))%>%

filter(!is.na(population2))

naive_or_oral <- for_text%>%

filter(population2 == "Drug naive" | population2 == "On oral therapy")

summary(as.factor(for_text2$population2))

older_naive_or_oral <- for_text2%>%

filter(population2 == "Drug naive" | population2 == "On oral therapy")

fi_cutoff_multiple <- function(y){

df <- data.frame(study = dist$nct_id[y],

above_0.1 = (1-pgengamma(q = 0.1,

mu = dist$mu[y],

sigma = dist$sigma[y],

Q = dist$Q[y]))*100,

above_0.2 = (1-pgengamma(q = 0.2,

mu = dist$mu[y],

sigma = dist$sigma[y],

Q = dist$Q[y]))*100,

above_0.3 = (1-pgengamma(q = 0.3,

mu = dist$mu[y],

sigma = dist$sigma[y],

Q = dist$Q[y]))*100,

above_0.4 = (1-pgengamma(q = 0.4,

mu = dist$mu[y],

sigma = dist$sigma[y],

Q = dist$Q[y]))*100)

df

}

supplementary_table <- map_df(1:34, fi_cutoff_multiple)%>%

left_join(test5%>%distinct(study, population2, mean_age))%>%

filter(!is.na(population2))

supplementary_table2 <- supplementary_table%>%

mutate(a = round(100-above_0.1, 2),

b = round(above_0.1-above_0.2, 2),

c = round(above_0.2-above_0.3, 2),

d = round(above_0.3-above_0.4, 2),

e = round(above_0.4, 2))%>%

select(population2, study, a:e)%>%

arrange(population2)

write.csv(supplementary_table2, "cut_point_table_corrected.csv")

library(rms)

describe(supplementary_table$above_0.2)

describe(supplementary_table$above_0.3)

describe(supplementary_table$above_0.4)

fi_cutoff_multiple2 <- function(y){

df <- data.frame(study = dist2$nct_id[y],

above_0.1 = (1-pgengamma(q = 0.1,

mu = dist2$mu[y],

sigma = dist2$sigma[y],

Q = dist2$Q[y]))*100,

above_0.2 = (1-pgengamma(q = 0.2,

mu = dist2$mu[y],

sigma = dist2$sigma[y],

Q = dist2$Q[y]))*100,

above_0.3 = (1-pgengamma(q = 0.3,

mu = dist2$mu[y],

sigma = dist2$sigma[y],

Q = dist2$Q[y]))*100,

above_0.4 = (1-pgengamma(q = 0.4,

mu = dist2$mu[y],

sigma = dist2$sigma[y],

Q = dist2$Q[y]))*100)

df

}

supplementary_table_65 <- map_df(1:34, fi_cutoff_multiple2)%>%

left_join(test5%>%distinct(study, population2, mean_age))%>%

filter(!is.na(population2))

describe(supplementary_table_65$above_0.2)

describe(supplementary_table_65$above_0.3)

# meta-analyses of adverse event data #####

# read in model estimates

events <- read_csv("adverse_event_models_corrected.csv")

# select relevant model then run random effects meta analysis with inverse variance weighting

ae <- events%>%

filter(model == "Adverse events")%>%

left_join(dist%>%distinct(nct_id, study))

ae_res <- metagen(TE = ae$est,

seTE = ae$se,

studylab = ae$nct_id,

sm = "RR")

sae <- events%>%

filter(model == "Serious adverse events")

sae_res <- metagen(TE = sae$est,

seTE = sae$se,

studylab = sae$study,

sm = "RR")

hypo <- events%>%

filter(model == "Hypoglycaemia events")

hypo_res <- metagen(TE = hypo$est,

seTE = hypo$se,

studylab = hypo$study,

sm = "RR")

attr <- events%>%

filter(model == "Attrition")

attr_res <- metagen(TE = attr$est,

seTE = attr$se,

studylab = attr$study,

sm = "OR")

# generate text for forest plots and plot each model

tabletext_ae <- cbind(c("Trial", NA, ae$nct_id, NA, "Summary"),

c(" ", NA, rep(NA, length(ae$nct_id)), NA, NA),

c("IRR", NA,

round(exp(ae_res$TE),2),

NA,

round(exp(ae_res$TE.random),2)),

c("95% CI", NA,

paste0("(", round(exp(ae_res$lower),2), " to ", round(exp(ae_res$upper),2), ")"),

NA,

paste0("(", round(exp(ae_res$lower.random),2), " to ", round(exp(ae_res$upper.random),2), ")") ))

fp_ae <- forestplot(labeltext = tabletext_ae,

mean = c(NA, NA, exp(ae_res$TE), NA, exp(ae_res$TE.random)),

lower = c(NA, NA, exp(ae_res$lower), NA,exp(ae_res$lower.random)),

upper = c(NA, NA, exp(ae_res$upper), NA, exp(ae_res$upper.random)),

txt_gp = fpTxtGp(label = gpar(fontfamily = "", cex=0.8)),

is.summary = c(T, F, rep(F, length(ae_res$TE)), F, T),

graphwidth = unit(2, "in"),

xlog = TRUE,

xticks = c(0.5, 1, 2, 4, 8, 16),

clip = c(0.5, 16),

boxsize = 0.5,

title = "Adverse events",

xlab = "Incidence rate ratio")

tabletext_sae <- cbind(c("IRR", NA,

round(exp(sae_res$TE),2),

NA,

round(exp(sae_res$TE.random),2)),

c("95% CI", NA,

paste0("(", round(exp(sae_res$lower),2), " to ", round(exp(sae_res$upper),2), ")"),

NA,

paste0("(", round(exp(sae_res$lower.random),2), " to ", round(exp(sae_res$upper.random),2), ")") ))

fp_sae <- forestplot(labeltext = tabletext_sae,

mean = c(NA, NA, exp(sae_res$TE), NA, exp(sae_res$TE.random)),

lower = c(NA, NA, exp(sae_res$lower), NA,exp(sae_res$lower.random)),

upper = c(NA, NA, exp(sae_res$upper), NA, exp(sae_res$upper.random)),

txt_gp = fpTxtGp(label = gpar(fontfamily = "", cex=0.8)),

is.summary = c(T, F, rep(F, length(sae_res$TE)), F, T),

graphwidth = unit(1.5, "in"),

xlog = TRUE,

xticks = c(0.5, 1, 2, 4, 8, 16),

clip = c(0.5, 16),

boxsize = 0.5,

title = "Serious adverse events",

xlab = "Incidence rate ratio")

tabletext_hypo <- cbind(c("IRR", NA,

round(exp(hypo_res$TE),2),

NA,

round(exp(hypo_res$TE.random),2)),

c("95% CI", NA,

paste0("(", round(exp(hypo_res$lower),2), " to ", round(exp(hypo_res$upper),2), ")"),

NA,

paste0("(", round(exp(hypo_res$lower.random),2), " to ", round(exp(hypo_res$upper.random),2), ")") ))

fp_hypo <- forestplot(labeltext = tabletext_hypo,

mean = c(NA, NA, exp(hypo_res$TE), NA, exp(hypo_res$TE.random)),

lower = c(NA, NA, exp(hypo_res$lower), NA,exp(hypo_res$lower.random)),

upper = c(NA, NA, exp(hypo_res$upper), NA, exp(hypo_res$upper.random)),

txt_gp = fpTxtGp(label = gpar(fontfamily = "", cex=0.8)),

is.summary = c(T, F, rep(F, length(hypo_res$TE)), F, T),

graphwidth = unit(1.5, "in"),

xlog = TRUE,

xticks = c(0.5, 1, 2, 4, 8, 16),

clip = c(0.5, 16),

boxsize = 0.5,

title = "Hypoglycaemic events",

xlab = "Incidence rate ratio")

tabletext_attr <- cbind(c("OR", NA,

round(exp(attr_res$TE),2),

NA,

round(exp(attr_res$TE.random),2)),

c("95% CI", NA,

paste0("(", round(exp(attr_res$lower),2), " to ", round(exp(attr_res$upper),2), ")"),

NA,

paste0("(", round(exp(attr_res$lower.random),2), " to ", round(exp(attr_res$upper.random),2), ")") ))

fp_attr <- forestplot(labeltext = tabletext_attr,

mean = c(NA, NA, exp(attr_res$TE), NA, exp(attr_res$TE.random)),

lower = c(NA, NA, exp(attr_res$lower), NA,exp(attr_res$lower.random)),

upper = c(NA, NA, exp(attr_res$upper), NA, exp(attr_res$upper.random)),

txt_gp = fpTxtGp(label = gpar(fontfamily = "", cex=0.8)),

is.summary = c(T, F, rep(F, length(attr_res$TE)), F, T),

graphwidth = unit(1.5, "in"),

xlog = TRUE,

xticks = c(0.5, 1, 2, 4, 8, 16),

clip = c(0.5, 16),

boxsize = 0.5,

title = "Attrition",

xlab = "Odds ratio")

# assessing differences FI-adverse event association by arm #####

dat <- read_csv("adverse_event_models_by_arm_corrected.csv")

arms <- read_csv("Export_13_3_24/efficacy_models_locf.csv")

test_by_arm <- function(data, arms_data, outcome){

dat1 <- data%>%

filter(model == outcome,

term == "I(FI * 10)",

!is.na(arm_drug))%>%

left_join(arms_data%>%distinct(study, arm_drug, n))

dat1 <- dat1%>%

mutate(class = case_when(arm_drug == "Canagliflozin" ~ "SGLT2",

arm_drug == "Dulaglutide" ~ "GLP1",

arm_drug == "Empagliflozin" ~ "SGLT2",

arm_drug == "Empagliflozin + Metformin" ~ "SGLT2",

arm_drug == "Exenatide" ~ "GLP1",

arm_drug == "Glimepiride" ~ "Sulphonylurea",

arm_drug == "Insulin" ~ "Insulin",

arm_drug == "Linagliptin" ~ "DPP4",

arm_drug == "Linagliptin + Metformin"~ "DPP4",

arm_drug == "Liraglutide" ~ "GLP1",

arm_drug == "Lixisenatide" ~ "GLP1",

arm_drug == "Metformin" ~ "Metformin",

arm_drug == "Placebo" ~ "Placebo",

arm_drug == "Sitagliptin" ~ "DPP4"))

nwork <- set_agd_arm(dat1, study = study, y = exp(est), se = exp(se), sample_size = n, trt = arm_drug, trt_class = class)

res <- nma(nwork, trt_effects = "random", cores = 2)

xmn1 <- summary(res)$summary

nwork_class <- set_agd_arm(dat1,

study = study, y = exp(est), se = exp(se),

sample_size = n, trt = class)

res_class <- nma(nwork_class, trt_effects = "random", cores = 2)

xmn <- summary(res_class)$summary

drug_effects <- xmn1%>%

filter(grepl("d", parameter))%>%

filter(!grepl("delta", parameter))%>%

mutate(parameter = str_sub(parameter, 2L, -1L))%>%

mutate(parameter = str_replace_all(parameter, "[[:punct:]]", ""))

class_effects <- xmn%>%

filter(grepl("d", parameter))%>%

filter(!grepl("delta", parameter))%>%

mutate(parameter = str_sub(parameter, 2L, -1L))%>%

mutate(parameter = str_replace_all(parameter, "[[:punct:]]", ""))

estimates <- drug_effects%>%

mutate(network = "Drug-level estimates")%>%

full_join(class_effects%>%mutate(network = "Class-level estimates"))

ggplot(estimates, aes(x = exp(mean), y = parameter, xmin = exp(`2.5%`), xmax =exp(`97.5%`)))+

geom_pointrange()+

geom_vline(xintercept = 1, linetype="dashed",

size=0.5)+

scale_x_continuous(trans = "log10")+

#facet_grid(network~., scales = "free_y")

facet_wrap(~network, ncol = 1, scales = "free_y")+

labs(title = "Frailty treatment interactions",

x = "Frailty-treatment interaction",

y = "Comparison (vs placebo)")

}

test_by_arm(data = dat, arms_data = arms, outcome = "Adverse events")

test_by_arm(data = dat, arms_data = arms, outcome = "Attrition")

test_by_arm(data = dat, arms_data = arms, outcome = "Serious adverse events")

# analysis of efficacy #####

library(tidyverse)

library(multinma)

library(truncnorm)

library(Matrix)

## Functions ----

CnvrtCorrMatrix <- function(a){

## recovery whole matrix by duplication

allnames <- union(a$row, a$col)

a <- bind_rows(a,

a %>%

rename(row = col, col = row),

tibble(row = allnames, col = allnames, r = 1)) %>%

distinct()

# convert into matrix format

a <- a %>%

spread(col, r)

a_row <- a$row

a$row <- NULL

a <- as.matrix(a)

if (any(is.na(a))) warning("Missing values in matrix")

rownames(a) <- a_row

a

}

## read in coefficients and variance/covariance matrix ----

mace <- list(cfs = read_csv("Data/fi_mace_model_coefs.csv"),

vcv = read_csv("Data/fi_mace_model_vcov.csv"),

diag = read_csv("Data/fi_mace_model_diags.csv"))

hba1c_main <- list(cfs = read_csv("Data/hb1c_fi_mod_coefs.csv") %>%

select(-study2) %>%

filter(!models == "b1"),

vcv = read_csv("Data/hb1c_fi_mod_vcov.csv") %>%

select(-study2) %>%

filter(!models == "b1"),

diag = read_csv("Data/hb1c_fi_mod_diags.csv") %>%

select(-study2)%>%

filter(!models == "b1"))

hba1c_sens <- list(cfs = read_csv("Data/hb1c_fi_mod_focf_coefs.csv") %>% select(-study2)%>%

filter(!models == "b1"),

vcv = read_csv("Data/hb1c_fi_mod_focf_vcov.csv") %>% select(-study2)%>%

filter(!models == "b1"),

diag = read_csv("Data/hb1c_fi_mod_focf_diags.csv") %>% select(-study2)%>%

filter(!models == "b1"))

tot <- list(mace = mace,

hba1c_main = hba1c_main,

hba1c_sens = hba1c_sens)

tot <- transpose(tot)

tot <- map(tot, ~ bind_rows(.x, .id = "outcome"))

cfs <- tot$cfs

cfs <- cfs %>%

select(outcome, study, models, term, estimate, se = std.error)

cfs <- cfs %>%

filter(!term %in% c("baseline_value", "(Intercept)"))

vcv <- tot$vcv

rm(tot)

refdrugs <- read_csv("Data/reference_arms.csv")

## drop combinations as this amounts to two arms with same dc in tria;

cfs <- cfs %>%

filter(!str_detect(term, "\\+"))

vcv <- vcv %>%

filter(!str_detect(row, "\\+"),

!str_detect(col, "\\+"))

## rename drugs to classes ----

## drop interaction terms, also drop arms with "+" metformin

smplft <- read_csv("trt,dc

Canagliflozin,SGLT2

Dulaglutide,GLP1

Empagliflozin,SGLT2

Empagliflozin + Metformin,SGLT2

Exenatide,GLP1

Glimepiride,Sulphonylurea

Insulin,Insulin

Linagliptin,DPP4

Linagliptin + Metformin,DPP4

Liraglutide,GLP1

Lixisenatide,GLP1

Metformin,Metformin

Placebo,Placebo

Sitagliptin,DPP4")

## replace all terms with dc terms and drop duplicates

## use superassigment

res <- cfs$term

walk2(smplft$trt, smplft$dc, ~ {

res <<- res %>%

str_replace(.x, .y) %>%

str_remove("arm_f_2_")

})

cfs <- cfs %>%

mutate(term_replace = res)

trm_lkp <- cfs %>%

distinct(term, term_replace)

trm_lkp_vct <- trm_lkp$term_replace

names(trm_lkp_vct) <- trm_lkp$term

cfs <- cfs %>%

rename(term_orig = term) %>%

rename(term = term_replace)

## join reference as extra column to allow to drop duplicate arms

refdrugs <- refdrugs %>%

filter(ref ==1)

refdrugs <- refdrugs %>%

select(study, trt = arm_drug) %>%

inner_join(smplft) %>%

select(study, reference = dc)

cfs <- cfs %>%

inner_join(refdrugs)

## pull treatment term out of full term

cfs <- cfs %>%

mutate(trt = str_extract(term, paste(smplft$dc, collapse = "|")))

## drop where the treatment is the same as the reference, retain NAs

cfs <- cfs %>%

filter(is.na(trt) | !reference == trt)

## take distinct arms; assumes that the ordering was the same for

cfs <- cfs %>%

distinct(outcome, study, models, term, reference, trt, .keep_all = TRUE)

# vcv <- vcv %>%

# mutate(across(c(row, col), ~ trm_lkp_vct[.x]))

## combine data and make sure correlation matrix aligns with terms

cfs <- cfs %>%

group_by(outcome, study, models) %>%

nest() %>%

ungroup()

vcv <- vcv %>%

group_by(outcome, study, models) %>%

nest() %>%

ungroup()

vcv2 <- cfs %>%

anti_join(vcv %>% select(-data))

vcv2$coef <- map_chr(vcv2$data, ~ .x %>% pull(term_orig))

vcv2$data <- map(vcv2$coef, ~ tibble(row = .x, col = .x, r = 1))

vcv2$coef <- NULL

vcv <- bind_rows(vcv, vcv2)

rm(vcv2)

## join cfs and vcv in a single tibble . one row per outcome, model and study

x <- cfs %>%

rename(cfs = data) %>%

inner_join(vcv %>% rename(vcv = data))

rm(cfs, vcv)

## rename and map vcv so matches cfs

# first convert to matrix

x$r <- map(x$vcv, CnvrtCorrMatrix)

## align rows and columns of matrices with coefficients and rename to new terms

x$r <- map2(x$r, x$cfs, ~ {

a <- .x [.y$term_orig, .y$term_orig, drop = FALSE]

colnames(a) <- .y$term

rownames(a) <- .y$term

a

})

## set up covariates as per set_agd_regression

x$cfs <- map2(x$cfs, x$models, ~ {

if(.y == "f1" ){

a <- .x

} else if(.y == "f2" ) {

a <- .x %>%

mutate(frail = if_else(str_detect(term, "FI10"), 1L, NA_integer_))

} else if (.y == "f3" ) {

a <- .x %>%

mutate(age = if_else(str_detect(term, "AGE"), 1L, NA_integer_),

male = if_else(str_detect(term, "SEXM"), TRUE, NA),

frail = if_else(str_detect(term, "FI10"), 1L, NA_integer_))

}

a})

# add in reference category ----

x$cfs <- map(x$cfs, function(a) {

a1 <- a %>%

slice(1) %>%

mutate(across(c(estimate, se), ~ NA_real_),

trt = reference,

across(c(term, term_orig), ~ "reference_row"))

a1[ , names(a1) %in% c("frail", "male", "age")] <- 0

bind_rows(a1, a)

})

cfs_rv <- x %>%

select(outcome, models, cfs, study) %>%

unnest(cfs) %>%

group_by(outcome, models, term, trt, age, male, frail) %>%

summarise(studies = sum(!duplicated(study)),

study_list = paste(study, models, sep = ", ", collapse = ";")) %>%

ungroup() %>%

arrange(outcome, models)

write_csv(cfs_rv, "Outputs/check_term_recoding.csv")

## crename trt to arm_lbl

x$cfs <- map(x$cfs, ~ .x %>%

rename(arm_lvl = trt) %>%

mutate(dc = case_when(

arm_lvl %in% c("DPP4", "GLP1", "SGLT2", "Metformin", "Sulphonylurea") ~ "oad",

arm_lvl %in% "Insulin" ~ "Insulin",

arm_lvl == "Placebo" ~ "Placebo",

is.na(arm_lvl) ~ NA_character_)))

## need to drop "H9X-MC-GBDE" as all arms are in the same dc

x <- x %>%

filter(!study =="H9X-MC-GBDE")

## check if any trials with multiple identical classes

x$duparms <- map_lgl(x$cfs, ~ .x %>%

filter(!is.na(term), !is.na(arm_lvl)) %>%

filter(!str_detect(term, "\\:")) %>%

summarise(dups = any(duplicated(arm_lvl))) %>%

pull(dups))

## check matrices still match terms, note drop first term as this is the reference row where est and se are set to NA

x_chk <- map2_lgl(x$cfs, x$r, function(cfs, r) {

all(cfs$term[-1] == colnames(r))

})

if(! all(x_chk)) stop("Terms do not match matrices")

## Rearrange data into outcome/model combinations so can have a list with each dataset ----

formod <- x %>%

nest(.by = c(outcome, models))

## simplify data structure

formod$data <- map(formod$data, ~ {

cfs <- .x %>%

select(study, cfs) %>%

unnest(cfs)

r <- .x$r

names(r) <- .x$study

list(cfs = cfs, r = r)

})

## Add in fomrulae ----

forms_mace <- tibble(models = paste0("f", 1:3),

forms = list(~ .trt,

~ frail*.trt,

~ (frail + male + age)*.trt))

forms_hba1c <- tibble(models = paste0("f", 1:3),

forms = list(~ .trt,

~ frail*.trt,

~ (frail + male + age)*.trt))

## note deliberately duplicating formuals for hba1c

forms <- bind_rows(mace = forms_mace,

hba1c_main = forms_hba1c,

hba1c_sens = forms_hba1c,

.id = "outcome")

formod <- formod %>%

inner_join(forms)

saveRDS(formod, "mace_hba1c_formod.Rds")

## supply using Rscript

args <- commandArgs(trailingOnly=TRUE)

i <- as.integer(args[[1]])

print(i)

mydfs <- readRDS("mace_hba1c_formod.Rds")

print(unlist(mydfs[i, c("outcome", "models")]))

mydata <- mydfs$data[[i]]$cfs

print(class(mydata))

mycor <- mydfs$data[[i]]$r

print(class(mycor))

print(class(mycor[[1]]))

myreg <- mydfs$forms[[i]]

print(class(myreg))

nwork <- set_agd_regression(mydata,

study = study,

trt = arm_lvl,

estimate = estimate,

se = se,

cor = mycor,

trt_ref = "Placebo",

trt_class = dc,

regression = myreg)

mdl_fe <- nma(nwork,

trt_effects = "fixed",

link = "identity",

regression = myreg,

class_interactions = "independent",

prior_intercept = normal(scale = 10),

prior_trt = normal(scale = 10),

prior_reg = normal(scale = 10),

chains = 4, cores = 4,

control = list(max_treedepth = 15))

saveRDS(mdl_fe, paste0("fe_fi_class_lvl", i, ".Rds"))

mdl_re <- nma(nwork,

trt_effects = "random",

link = "identity",

regression = myreg,

class_interactions = "independent",

prior_intercept = normal(scale = 10),

prior_trt = normal(scale = 10),

prior_reg = normal(scale = 10),

chains = 4, cores = 4,

control = list(max_treedepth = 15))

saveRDS(mdl_re, paste0("re_fi_class_lvl", i, ".Rds"))

library(tidyverse)

library(multinma)

library(rstan)

modelfiles <- list.files("FromVM/", patt = "Rds$")

res <- map(modelfiles, ~ readRDS(paste0("FromVM/", .x)))

modeln <- str_extract(modelfiles, "[0-9]")

fere <- str_sub(modelfiles, 1, 2)

nm <- paste0(fere, modeln)

smry <- map(res, ~ summary(.x)$summary)

names(smry) <- nm

smpls <- map(res, ~ as.data.frame(.x)$stanfit)

names(smpls) <- nm

divergent <- map(res, ~ rstan::get_sampler_params(.x$stanfit, inc_warmup = FALSE))

divergent <- map(divergent, ~ map(.x, as_tibble))

divergent <- map(divergent, ~ bind_rows(.x, .id = "chain"))

names(divergent) <- nm

divergent <- bind_rows(divergent, .id = "model")

saveRDS(list(smry = smry, smpls = smpls, divergent = divergent), "smry_mdls.Rds")

rm(res)

# shinystan::launch_shinystan(fe_cls$stanfit)

smry_mdls <- readRDS("smry_mdls.Rds")

all_models <- smry_mdls[[1]]

names(all_models)

re_hba <- all_models[[13]]

# trials <- re_hba%>%

# filter(grepl("mu", parameter))%>%

# mutate(parameter = str_replace_all(parameter, "[[:punct:]]", ""))%>%

# mutate(parameter = str_replace_all(parameter, "mu", ""))

res_interaction_hba <- re_hba%>%

filter(grepl("beta", parameter))%>%

mutate(parameter = str_sub(parameter, 5L, -1L))%>%

mutate(parameter = str_replace_all(parameter, "[[:punct:]]", ""))%>%

filter(parameter %in% c("trtDPP4frail", "trtSGLT2frail", "trtGLP1frail"))%>%

mutate(parameter = gsub("trt", "", parameter))%>%

mutate(parameter = gsub("frail", "", parameter))%>%

mutate(analysis = "HbA1c (%)")

res_main_effect_hba <- re_hba%>%

filter(grepl("d", parameter))%>%

filter(!grepl("delta", parameter))%>%

mutate(parameter = str_replace_all(parameter, "[[:punct:]]", ""))%>%

mutate(parameter = gsub("d", "", parameter))%>%

filter(parameter %in% c("DPP4", "SGLT2", "GLP1"))%>%

mutate(analysis = "HbA1c (%)")

res_all_hba <- res_interaction_hba%>%

mutate(effect = "Frailty-treatment interaction")%>%

bind_rows(res_main_effect_hba%>%

mutate(effect = "Main effect"))

ggplot(res_all_hba,

aes(x = mean, y = parameter, xmin = `2.5%`, xmax =`97.5%`, colour = effect))+

geom_point(position = position_dodge(width = .5)) +

geom_linerange(position = position_dodge(width = .5)) +

geom_vline(xintercept = 0, linetype="dashed",

size=0.5)+

facet_wrap(~analysis, ncol = 1, scales = "free_y")+

labs(x = "Frailty-treatment interaction per 0.1-point increase in frailty index",

y = "Treatment")

re_mace <- all_models[[9]]

res_interaction_mace <- re_mace%>%

filter(grepl("beta", parameter))%>%

mutate(parameter = str_sub(parameter, 5L, -1L))%>%

mutate(parameter = str_replace_all(parameter, "[[:punct:]]", ""))%>%

filter(parameter %in% c("trtDPP4frail", "trtSGLT2frail", "trtGLP1frail"))%>%

mutate(parameter = gsub("trt", "", parameter))%>%

mutate(parameter = gsub("frail", "", parameter))%>%

mutate(analysis = "MACE (log hazard ratio)")

res_main_effect_mace <- re_mace%>%

filter(grepl("d", parameter))%>%

filter(!grepl("delta", parameter))%>%

mutate(parameter = str_replace_all(parameter, "[[:punct:]]", ""))%>%

mutate(parameter = gsub("d", "", parameter))%>%

filter(parameter %in% c("DPP4", "SGLT2", "GLP1"))%>%

mutate(analysis = "MACE (log hazard ratio)")

res_all_mace <- res_interaction_mace%>%

mutate(effect = "Frailty-treatment interaction")%>%

bind_rows(res_main_effect_mace%>%

mutate(effect = "Main effect"))

ggplot(res_all_mace,

aes(x = mean, y = parameter, xmin = `2.5%`, xmax =`97.5%`, colour = effect))+

geom_point(position = position_dodge(width = .5)) +

geom_linerange(position = position_dodge(width = .5)) +

geom_vline(xintercept = 0, linetype="dashed",

size=0.5)+

facet_wrap(~analysis, ncol = 1, scales = "free_y")+

labs(x = "Frailty-treatment interaction per 0.1-point increase in frailty index",

y = "Treatment")

res_all <- res_all_hba%>%

bind_rows(res_all_mace)

ggplot(res_all,

aes(x = mean, y = parameter, xmin = `2.5%`, xmax =`97.5%`, colour = effect))+

geom_point(position = position_dodge(width = .5)) +

geom_linerange(position = position_dodge(width = .5)) +

geom_vline(xintercept = 0, linetype="dashed",

size=0.5)+

facet_wrap(~analysis, ncol = 1, scales = "free_y")+

labs(x = "Frailty-treatment interaction per 0.1-point increase in frailty index",

y = "Treatment")
