# Supplementary material for: Frailty in randomized controlled trials of glucose-lowering therapies for type 2 diabetes: An individual participant data meta-analysis of frailty prevalence, treatment efficacy, and adverse events
Source: PLoS Med. 2025 Apr 7;22(4):e1004553. doi: 10.1371/journal.pmed.1004553 (PMC12052138; doi:10.1371/journal.pmed.1004553)
Supplement: S1 Table — (DOCX) [file pmed.1004553.s003.docx]

|  | Dipeptidyl peptidase 4 (DPP-4) inhibitors aggregate | Dipeptidyl peptidase 4 (DPP-4) inhibitors IPD (all) | Dipeptidyl peptidase 4 (DPP-4) inhibitors IPD (included in frailty analysis) | Glucagon-like peptide-1 (GLP-1) analogues aggregate | Glucagon-like peptide-1 (GLP-1) analogues IPD (all) | Glucagon-like peptide-1 (GLP-1) analogues IPD (included in frailty analysis) | Sodium-glucose co-transporter 2 (SGLT2) inhibitors aggregate | Sodium-glucose co-transporter 2 (SGLT2) inhibitors IPD (all) | Sodium-glucose co-transporter 2 (SGLT2) inhibitors IPD (included in frailty analysis) | Total trials aggregate | Total trials IPD (all) | Total trials IPD (included in frailty analysis) |
| --- | --- | --- | --- | --- | --- | --- | --- | --- | --- | --- | --- | --- |
| Trials | 237 | 26 | 17 | 158 | 25 | 9 | 140 | 22 | 10 | 489 | 70 | 34 |
| 2 arms | 204 | 13 | 14 | 106 | 19 | 3 | 107 | 10 | 2 | 388 | 38 | 19 |
| 3 arms | 25 | 10 |  | 41 | 4 | 5 | 28 | 11 | 5 | 80 | 24 | 10 |
| 4 or 5 arms | 8 | 3 | 3 | 11 | 2 | 1 | 5 | 1 | 3 | 21 | 8 | 5 |
| Participants | 109293 | 19530 | 10461 | 79184 | 21531 | 6606 | 44039 | 31425 | 8766 | 217321 | 69287 | 25208 |
| Male | 63066 (57.7%) | 11072 (56.7%) | 5652 (54.0%) | 44780 (56.6%) | 12788 (59.4%) | 3521 (53.3%) | 24776 (56.3%) | 19954 (63.5%) | 4684 (53.4%) | 124159 (57.1%) | 42186 (60.9%) | 13511 (53.6%) |
| Age | 58.8 (10.8) [40.4-75.7] | 57.0 (11.3) [36.7-74.9] | 57.6 (11.2) [37.2-75.4] | 57.9 (10.3) [40.5-74.4] | 59.7 (11.0) [40.5-76.4] | 58.0 (10.9) [38.3-75.4] | 61.5 (10.8) [43.0-78.3] | 58.2 (11.2) [36.5-75.6] | 56.1 (10.9) [36.1-74.0] | 59.2 (10.7) [41.0-76.0] | 58.5 (11.2) [37.7-75.7] | 57.5 (11.1) [37.3-75.2] |
| Duration | 24.0 (12.0-54.4) | 25.0 (14.5-91.5) | 24.0 (12.0-52.0) | 26.0 (12.0-56.0) | 24.0 (24.0-52.0) | 26.0 (24.8-41.6) | 24.0 (12.0-52.0) | 26.0 (16.1-239.2) | 24.0 (20.7-80.6) | 24.0 (12.0-56.0) | 25.0 (14.9-167.4) | 24.0 (15.6-52.0) |

Table S1a of trial characteristics comparing all trials with data on HbA1c (excluding 9 trials with data on MACE only, none of which had access to individual participant data [IPD])

Table S1b of trial characteristics comparing all trials with data on HbA1c (excluding 9 trials with data on MACE only, none of which had access to individual participant data [IPD])

| nm | Dipeptidyl peptidase 4 (DPP-4) inhibitors aggregate | Dipeptidyl peptidase 4 (DPP-4) inhibitors IPD (all) | Dipeptidyl peptidase 4 (DPP-4) inhibitors IPD (included in frailty analysis) | Glucagon-like peptide-1 (GLP-1) analogues aggregate | Glucagon-like peptide-1 (GLP-1) analogues IPD (all) | Glucagon-like peptide-1 (GLP-1) analogues IPD (included in frailty analysis) | Sodium-glucose co-transporter 2 (SGLT2) inhibitors aggregate | Sodium-glucose co-transporter 2 (SGLT2) inhibitors IPD (all) | Sodium-glucose co-transporter 2 (SGLT2) inhibitors IPD (included in frailty analysis) | Total trials aggregate | Total trials IPD (all) | Total trials IPD (included in frailty analysis) |
| --- | --- | --- | --- | --- | --- | --- | --- | --- | --- | --- | --- | --- |
| Total | 237 | 26 | 17 | 158 | 9 | 25 | 140 | 22 | 10 | 489 | 70 | 34 |
| Placebo | 120 | 16 | 15 | 68 | 3 | 17 | 95 | 14 | 7 | 278 | 49 | 23 |
| Dipeptidyl peptidase 4 (DPP-4) inhibitors | - | - | - | 19 | 0 | 3 | 18 | 3 | 3 | 266 | 35 | 17 |
| Glucagon-like peptide-1 (GLP-1) analogues | 26 | 3 | 0 | - | - | - | 9 | 0 | 0 | 223 | 32 | 17 |
| Sodium-glucose co-transporter 2 (SGLT2) inhibitors | 19 | 4 | 5 | 9 | 0 | 0 | - | - | - | 175 | 37 | 18 |
| Sulfonylureas | 26 | 3 | 1 | 8 | 0 | 1 | 12 | 2 | 1 | 45 | 5 | 2 |
| INSULINS AND ANALOGUES | 5 | 0 | 0 | 40 | 4 | 4 | 1 | 0 | 0 | 44 | 4 | 4 |
| Biguanides | 23 | 7 | 2 | 4 | 1 | 0 | 3 | 1 | 2 | 29 | 8 | 5 |
| Thiazolidinediones | 15 | 0 | 0 | 5 | 0 | 1 | 4 | 0 | 0 | 22 | 1 | 0 |
| Alpha glucosidase inhibitors | 12 | 1 | 0 | 2 | 0 | 0 | 1 | 0 | 0 | 14 | 1 | 0 |
| BLOOD GLUCOSE LOWERING DRUGS, EXCL. INSULINS | 1 | 0 | 0 | 3 | 0 | 0 | 0 | 0 | 0 | 4 | 0 | 0 |
| Other blood glucose lowering drugs, excl. insulins | 2 | 0 | 0 | 0 | 0 | 0 | 0 | 0 | 0 | 2 | 0 | 0 |
