# Supplementary material for: Frailty in randomized controlled trials of glucose-lowering therapies for type 2 diabetes: An individual participant data meta-analysis of frailty prevalence, treatment efficacy, and adverse events
Source: PLoS Med. 2025 Apr 7;22(4):e1004553. doi: 10.1371/journal.pmed.1004553 (PMC12052138; doi:10.1371/journal.pmed.1004553)
Supplement: S2 Table — (DOCX) [file pmed.1004553.s004.docx]

Table S2 Percentage of trial participants and various frailty index thresholds, stratified by target population

| **Target population** | **Trial ID** | Frailty index (% participants) | | | | |
| --- | --- | --- | --- | --- | --- | --- |
|  |  | 0 to 0.1 | >0.1 to 0.2 | >0.2 to 0.3 | >0.3 to 0.4 | >0.4 |
| Drug Naïve | NCT00621140 | 64.84 | 32.92 | 2.16 | <0.1 | <0.1 |
|  | NCT00740051 | 52.52 | 42.05 | 5.05 | 0.36 | <0.1 |
|  | NCT01126580 | 31.41 | 53.56 | 13.97 | 1.03 | <0.1 |
|  | NCT01177813 | 61.23 | 36.37 | 2.35 | <0.1 | <0.1 |
|  | NCT01194830 | 36.42 | 54.25 | 8.76 | 0.55 | <0.1 |
|  | NCT01558271 | 71.37 | 28.02 | 0.61 | <0.1 | <0.1 |
|  | NCT01719003 | 54.02 | 41.98 | 3.8 | 0.2 | <0.1 |

| On oral therapy | NCT00601250 | 57.57 | 39.61 | 2.73 | <0.1 | <0.1 |
| --- | --- | --- | --- | --- | --- | --- |
|  | NCT00602472 | 56.96 | 41.21 | 1.82 | <0.1 | <0.1 |
|  | NCT00622284 | 51.59 | 45.04 | 3.3 | <0.1 | <0.1 |
|  | NCT00641043 | 63.51 | 34.39 | 2.03 | <0.1 | <0.1 |
|  | NCT00734474 | 41.94 | 48.44 | 8.9 | 0.69 | <0.1 |
|  | NCT00798161 | 74.68 | 24.54 | 0.77 | <0.1 | <0.1 |
|  | NCT00819091 | 50.91 | 43.56 | 5.2 | 0.32 | <0.1 |
|  | NCT00996658 | 65.18 | 33.05 | 1.71 | <0.1 | <0.1 |
|  | NCT01064687 | 26.39 | 53.99 | 17.4 | 2.08 | 0.13 |
|  | NCT01075282 | 32.65 | 51.87 | 13.93 | 1.47 | <0.1 |
|  | NCT01106625 | 33.56 | 51.66 | 13.54 | 1.2 | <0.1 |
|  | NCT01106677 | 36.56 | 51.61 | 11.04 | 0.77 | <0.1 |
|  | NCT01137812 | 30.77 | 52.07 | 14.98 | 2 | 0.18 |
|  | NCT01159600 | 60.78 | 36.88 | 2.28 | <0.1 | <0.1 |
|  | NCT01167881 | 54.62 | 43.3 | 2.06 | <0.1 | <0.1 |
|  | NCT01210001 | 55.75 | 40.88 | 3.28 | <0.1 | <0.1 |
|  | NCT01624259 | 32.6 | 52.08 | 14.08 | 1.19 | <0.1 |
|  | NCT01648582 | 77.53 | 21.07 | 1.31 | <0.1 | <0.1 |

| On insulin | NCT00954447 | 31.55 | 57.48 | 10.45 | 0.51 | <0.1 |
| --- | --- | --- | --- | --- | --- | --- |
|  | NCT01191268 | 17.99 | 55.26 | 23.91 | 2.74 | 0.1 |
|  | NCT01306214 | 29.6 | 60.83 | 9.28 | 0.29 | <0.1 |
|  | NCT01768559 | 25.76 | 55.99 | 16.44 | 1.72 | <0.1 |

| Older people (>70 years) | NCT01084005 | 28.54 | 57.16 | 13.02 | 1.21 | <0.1 |
| --- | --- | --- | --- | --- | --- | --- |
|  | NCT01798706 | 10.93 | 64.11 | 22.97 | 1.91 | <0.1 |

| Chronic renal impairment | NCT00800683 | <0.1 | 11.08 | 54.45 | 28.72 | 5.74 |
| --- | --- | --- | --- | --- | --- | --- |
|  | NCT01087502 | 2.97 | 32.5 | 46.85 | 16.18 | 1.49 |
|  | NCT01164501 | 17.71 | 59.33 | 20.9 | 1.99 | <0.1 |
