# Supplementary figures and images for: Frailty in randomized controlled trials of glucose-lowering therapies for type 2 diabetes: An individual participant data meta-analysis of frailty prevalence, treatment efficacy, and adverse events
Source: PLoS Med. 2025 Apr 7;22(4):e1004553. doi: 10.1371/journal.pmed.1004553 (PMC12052138; doi:10.1371/journal.pmed.1004553)

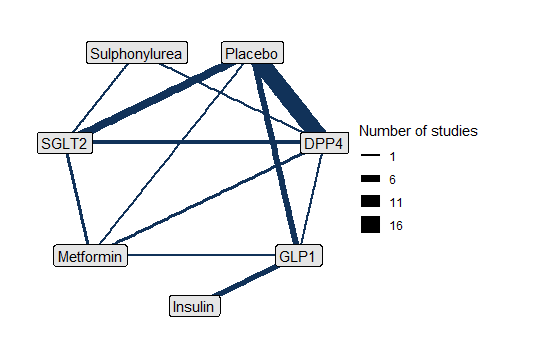

Supplement: S1 Fig — (PNG) [file pmed.1004553.s005.png]

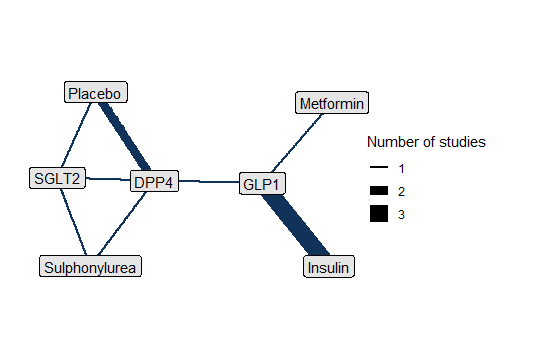

Supplement: S2 Fig — (PNG) [file pmed.1004553.s006.png]

# Frailty index distribution by age

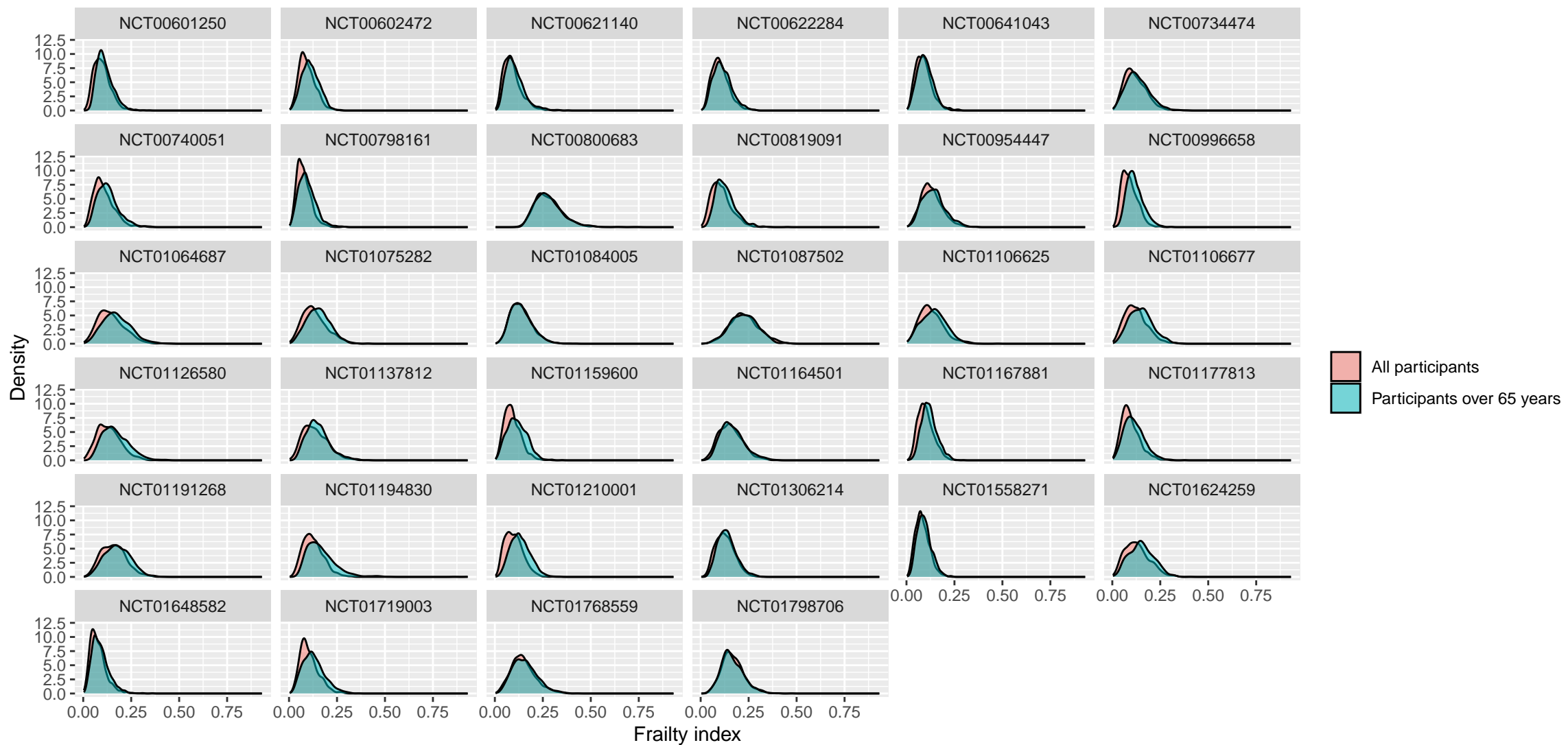

Supplement: S3 Fig — (PDF) [file pmed.1004553.s007.pdf]

# Frailty index distribution by sex

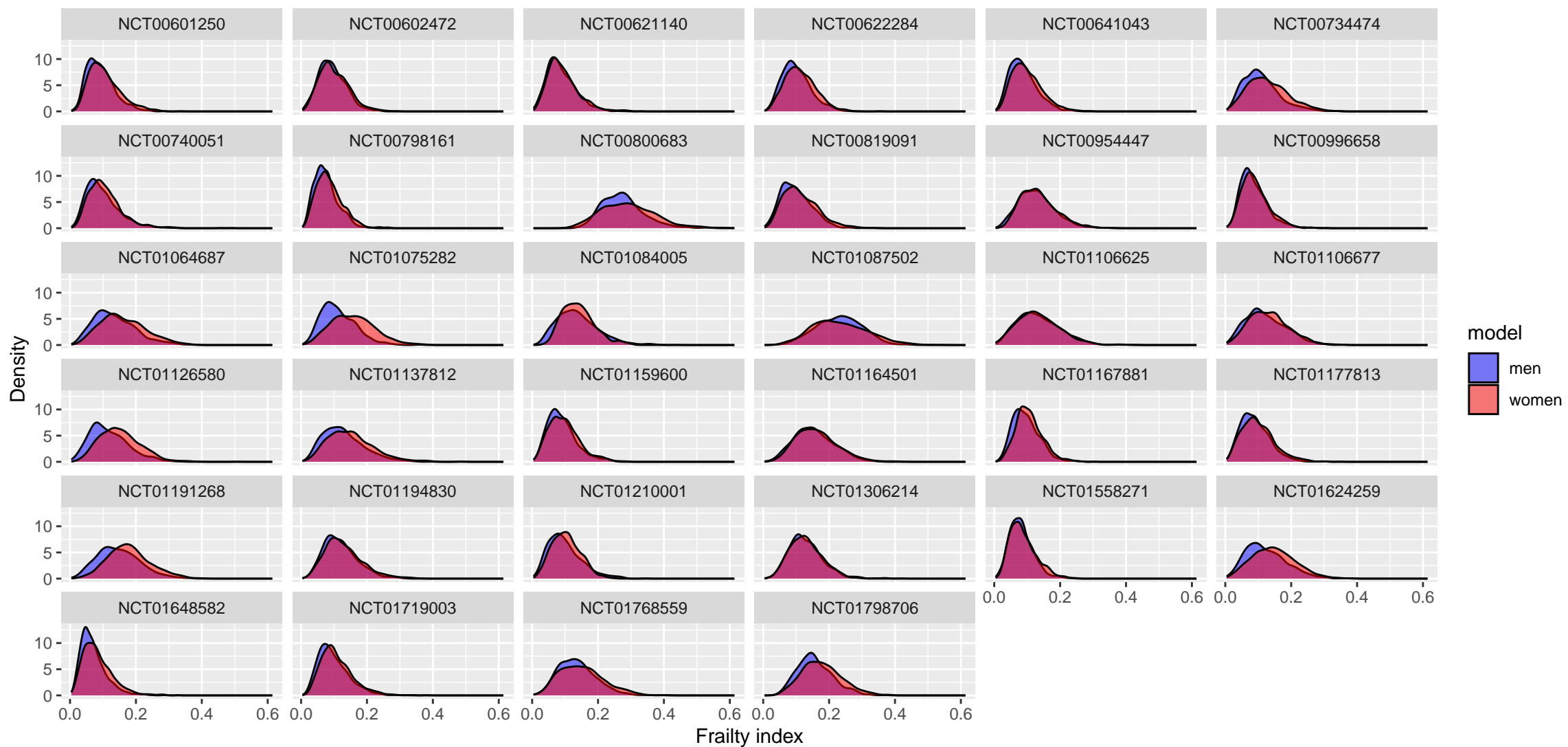

Supplement: S4 Fig — (PDF) [file pmed.1004553.s008.pdf]

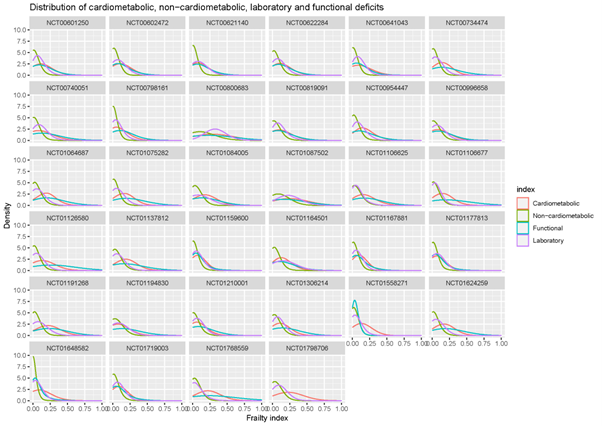

Supplement: S5 Fig — (PNG) [file pmed.1004553.s009.png]

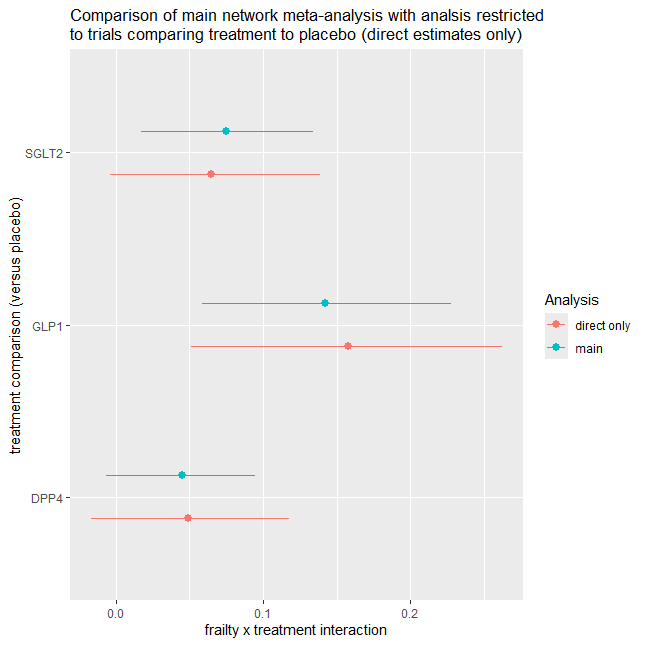

Supplement: S6 Fig — (PNG) [file pmed.1004553.s010.png]
