# Supplementary material for: Frailty in randomized controlled trials of glucose-lowering therapies for type 2 diabetes: An individual participant data meta-analysis of frailty prevalence, treatment efficacy, and adverse events
Source: PLoS Med. 2025 Apr 7;22(4):e1004553. doi: 10.1371/journal.pmed.1004553 (PMC12052138; doi:10.1371/journal.pmed.1004553)
Supplement: Attachment — Submitted filename: cover letter headed error correction.pdf [file pmed.1004553.s017.pdf]

27th March 2025

Dear Dr Van Epps,

Re: Frailty in randomised controlled trials of glucose lowering therapies for type 2 diabetes: An individual participant data meta-analysis of frailty prevalence, treatment efficacy, and adverse events.

Thank you for the opportunity to submit a corrected version the above manuscript. As you will be aware, following initial editorial acceptance, we identified a coding error within the analysis and requested some time to assess the impact of this on our study findings. We apologise for this genuine error, and are very grateful to the editorial team for allowing us to investigate this.

I am pleased to say that the error does not change the overall findings or conclusion of our manuscript. There are some small changes to the point estimates of frailty prevalence for some trials. I will outline here the nature of the error and how it occurred, as well as the changes made to the manuscript as a result.

Our study was based on a cumulative deficit frailty index: a cumulative total of the number of age-related health deficits present among trial participants (40-50 deficits per trial). The error related to the identification of hypertension (one of the included deficits) within a subset of the trials.

We identified a diagnosis of hypertension using medical history data coded using MedDRA codes (i.e. identifying relevant codes from the list of medical history items coded at baseline assessment). For some conditions, there was also an additional file which detailed selected medical conditions as binary variables (e.g. the presence or absence of hypertension). For 12 of the 34 included trials, we had inadvertently omitted the latter when identifying hypertension (i.e. participants were identified as having hypertension only if they had a relevant MedDRA code, but some participants who had hypertension coded from the binary data were misclassified as not having hypertension). The was a case of human error, having overlooked the second variable.

We have now re-run all analyses having corrected the specification of hypertension in these trials.

For some participants, their frailty index increased as a result (i.e. there was an additional deficit present). As such the estimates of frailty prevalence were slightly higher. However, these differences were small in magnitude and do not alter the interpretation of the results.

The results of the remaining analyses were unchanged.

We have highlighted all of the changes to the originally accepted version of the manuscript as tracked changes within the resubmission (alongside a clean version). We have also updated the supplementary figures (S3 and S4), supplementary tables (S2) and the supplementary data (including the corrected

results exported from the analysis platform allowing our analysis to be re-run using the supplementary files).

Yours sincerely

Dr Peter Hanlon
Clinical Senior Research Fellow
University of Glasgow

School of Health & Wellbeing
College of Medical, Veterinary and Life Sciences
University of Glasgow
1 Lilybank Gardens
G12 8RZ
T: +44(0)141 330 3299
E: shwadmin@glasgow.ac.uk
Tw: @UofGSHW

The University of Glasgow, charity number SC004401
