# Supplementary material for: Frailty in randomized controlled trials of glucose-lowering therapies for type 2 diabetes: An individual participant data meta-analysis of frailty prevalence, treatment efficacy, and adverse events
Source: PLoS Med. 2025 Apr 7;22(4):e1004553. doi: 10.1371/journal.pmed.1004553 (PMC12052138; doi:10.1371/journal.pmed.1004553)
Supplement: Attachment — Submitted filename: Response to reviewers.docx [file pmed.1004553.s015.docx]

Dear Dr Van Epps,

Thank you for the opportunity to submit a revision of our article, and to the reviewers for their comments and suggestions. Below we have outlined our response to each of the reviewers’ comments along with detail of all changes to the manuscript.

Yours sincerely

Dr Peter Hanlon, on behalf of the study team

Reviewer #1:
On careful reading of the paper I had no major methodological comments. I note that this is a first revision (I was not involved in the previous round of review). [Editors’ note: this reviewer appears to be referring to the ‘R1’ in the manuscript number; the editors will clarify that this was the first round of review, and there is no need to respond to this comment.]

The authors have described a very important evidence-practice gap. Increasingly our hospitals and primary care health centres are filled with older people living with frailty and yet, we have few reliable data to guide our treatment decisions. Historically, this was often due to clinical trials having upper age limited related to retirement age (e.g. 65 years), or due to erroneous reading of cholesterol epidemiology (with the historical age limit of 75 years). Despite recommendations that upper age limits should only be used if there was good evidence to suggest recruiting older people is definitely harmful, it remains surprising that these diabetic trials still had age limits.

I would recommend that the authors also include in the methods the original denominator of potential trials (as seen in Figure n = 596). Even with the 6% of trials with IPD available, less than half could contribute, and of those, remarkable few people living with frailty were recruited. The key message from this paper is that the frail have not been appropriately recruited into diabetes trials, and we therefore have substantial uncertainty of the benefits of many new treatments. Given that most people with diabetes are older people, and many have frailty (and corresponding co-morbidity, with the resulting polypharmacy) there is additional uncertainty whether the addition of these newer agents will have net harm or benefit.

Thank you. We have added a table to the supplementary appendix giving details of the wider body of trials, those for which we had IPD, and (of these trials) those that we were able to assess frailty. The following text has been added to the results to signpost this:

“The trials for which we could assess frailty were similar to the wider body of eligible trials in terms of mean age, sex distribution, treatments assessed and type of comparison (detailed in the supplementary appendix). However, while we identified 23 trials that were designed and powered to assess cardiovascular outcomes, and obtained IPD for 6 of these, none of these larger trials had sufficient data on function for us to assess frailty.”

We agree with the broader point about frailty only being estimable in a small subset of these trials, and that it was uncommon in these, and therefore the uncertainty around net benefits. We have expanded on this point in the discussion in response to the comment below.

I think the authors have missed an opportunity for a call to arms: the drug regulators and the government funders should particularly request data on frail older people, and new practical arrangements must be supported to allow the recruitment of those living with frailty. The authors quite rightly point out that one certain aspect of their paper is that the event rates are driven up with increasing frailty, so event driven trials will achieve outcomes quite quickly in this age group. The pharmaceutical industry is terrified that their nice new drug will be associated with unfortunate adverse events if used in those with frailty and even if not actively avoided, the actual practicalities of trial procedures will often lead to only the robust being able to manage intense trial monitoring of follow-up procedures. Older people with frailty can be recruited but you probably need more time for baseline assessment, you need to measure important aspects of health such as cognition
and function, you need frailty friendly trial procedures (such as home visits or remote assessment) and you need to ensure the procedures are not too onerous. Trials within cohorts, and the use of routinely collected data (as used, for example in the RECOVERY trials in Covid) provide examples of some work arounds. Frailty can also be electronically calculated with many electronic patient records.

Thank you for these comments. We agree with the reviewer’s assessment and have expanded the following section of the abstract and the discussion (new text in bold):

Abstract:

“While these findings are compatible with calls to relax HbA1c-based targets in people living with frailty, they also highlight the need for inclusion of people living with frailty in trials. **This would require changes to trial processes to facilitate the explicit assessment of frailty and support the participation of people living with frailty. Such changes are important as** the absolute balance of risks and benefits remains uncertain among those with higher degrees of frailty, who are largely excluded from trials.”

Discussion:
“**We need clinical evidence that is applicable to the populations who are being treated. Our findings demonstrate an important discrepancy between these trial populations (in which higher degrees of frailty were rare, even in trials focusing on older populations) and clinical practice (in which an increasing proportion of people with type 2 diabetes are living with frailty). We also show that it was only possible to assess frailty in a small subset of trials that assessed functional measures, and not in the larger cardiovascular outcome trials which are arguably most influential in terms of clinical practice and guidelines.** There is a need for trials including people with higher degrees of frailty, based on clinical outcomes which are a priority for patients. For this to happen, there is a need for trials to adopt inclusion and exclusion criteria that enable people living with frailty to take part; to measure a broad range of comorbidities and functional status to allow frailty to be reliably assessed **(and ideally including additional domains, such as cognition, that were not assessed in these trials);**^27^ to be designed to make it easy for older people living with frailty to participate **(including allowing sufficient time for detailed baseline assessments and adopting approaches to outcome assessment that minimise the burden on participants)**; and to measure clinical endpoints that are relevant to people living with frailty rather than solely assessing surrogate markers.^28^”

Overall, this is an important paper that illustrates a major evidence practice gap in the care of older people with frailty (a very large proportion of many hospitals worldwide). Richard Lindley. University of Sydney


Reviewer #2 (statistical review):
Firstly, I would like to thank the authors for their recent submission. This submission was an IPD analysis of frailties impacts on treatment, including adverse events. Overall, the work is well written and presented. I have some points below for your consideration:

1. Abstract: mean age is reported as a range. Is this the mean age across the included trials? Please clarify in text.

Thank you. We have edited this to the following to clarify:

“Mean age across the included trials ranged from 53·8 to 74·2 years.”

2. For the linear models (e.g. HbA1c), adjustments were made for baseline, age and sex. Did the authors consider use participant ID as a random factor in a mixed effect model? Or was this deemed not necessary due to the data type (e.g. no repeated measurements)? It may be worthwhile conducting a mixed model and comparing model fits between the current model and mixed model.

Thank you for this suggestion. In response to this comment we have fitted additional models for the HbA1c outcome (outcome variable) using age, sex and frailty index and independent variables and assessing interactions between each of these factors and treatment allocation, with respect to HbA1c.

Our original model used endpoint HbA1c as the outcome (with last observation carried forward for those participants who ceased follow-up prior to the primary endpoint), adjusting for baseline HbA1c (ANCOVA model) and including frailty x treatment, age x treatment and sex x treatment interaction terms.

We have now also fit a linear mixed effects model including all available HbA1c values over follow-up time, with a random intercept and slope for each participant and interaction terms for frailty and arm. We then compared the coefficients and standard errors from this model with the ANCOVA model from the primary analysis. The estimates of the frailty treatment interactions were similar for each analysis (the direction of effect was the same for all, point estimates were within 0.5 standard error for all but one estimate). We have not included visualizations of this comparison in the response/resubmission as this new analysis would require to be exported from the secure research environment and would then be subject to a 30-day review period by each of the trial sponsors. We can, of course, initiate this process should the editors/reviewers require this.

3. How was consistency between the direct and indirect evidence assessed?

We have assessed the consistency between direct and indirect evidence by repeating each network meta-analysis after restricting our dataset to the trials comparing the trial drug with placebo (and thus those providing direct estimated of frailty x treatment interactions compared to placebo, 23/34 trials). The frailty x treatment interactions for these models were consistent with the overall models (with slightly wider confidence intervals, see plots below). We have added the following text to the methods and results to highlight this.

“To assess consistency between direct and indirect estimates of the frailty x treatment interactions we fitted further network meta-analysis restricting the dataset to trials in which the comparator arm was placebo. We then compared these (direct) estimated to the overall estimate for which the comparator arms could include active treatment from the primary analysis.”

And

“These interaction terms were similar after limiting the analysis to trials the 23 trials with placebo comparisons (supplementary appendix).”

4. Heterogeneity is mentioned in the discussion, stating there was increased statistical power to assess heterogeneity. I could have missed the details in the text/supplementary material. I struggled to find evidence of this being reported. Could the authors please clarify in text what the heterogeneity assessment statistics were and the results obtained.

We were referring to heterogeneity in the sense of differences in treatment efficacy by individual level characteristics (i.e. heterogeneity in treatment efficacy, rather than assessment of heterogeneity among the included studies). We have re-worded this section to avoid ambiguity.

“Analyses of single trials are typically under-powered to detect **differences in treatment efficacy** by participant characteristics.... By combining 34 trials , our IPD network meta-analysis provides considerably greater statistical power to assess **differences in treatment efficacy** than previous analyses across individual-level characteristics, and found a small but statistically significant difference in efficacy on glycaemic outcomes by frailty.”

5. I would recommend the inclusion of GRADE for NMAs, see: [https://doi.org/10.1136/bmj-2022-074495](https://eur03.safelinks.protection.outlook.com/?url=https%3A%2F%2Fwww.bmj.com%2Fcontent%2F381%2Fbmj-2022-074495&data=05%7C02%7CPeter.Hanlon%40glasgow.ac.uk%7Ca3f0c118758741db9ebd08dd1dd8e843%7C6e725c29763a4f5081f22e254f0133c8%7C1%7C0%7C638699539637079528%7CUnknown%7CTWFpbGZsb3d8eyJFbXB0eU1hcGkiOnRydWUsIlYiOiIwLjAuMDAwMCIsIlAiOiJXaW4zMiIsIkFOIjoiTWFpbCIsIldUIjoyfQ%3D%3D%7C0%7C%7C%7C&sdata=oJqlWIKXATdeLvfEhnMBkkNwTl4QgNxrI5JzGqt1Pjo%3D&reserved=0)

6. There is no specific guidance for IPD NMAs, but this could aid in the interpretation of the results.

Combined response to points 5 and 6:

We thank the reviewer for highlighting this reference giving detail on the application of GRADE to network meta-analyses. While we agree with the importance of carefully assessing the certainty of the evidence presented (see additions below), this application of GRADE would not fit with the focus and aims of this paper, as the process is designed to assess the certainty of main treatment effects (drug A versus drug B). We are focused (in the efficacy analyses) on differences in treatment effects by frailty. These guidelines do not offer any recommendations for presenting or assessing the certainty of analysis of treatment effect heterogeneity (which is the focus of the efficacy analyses in this paper).

We would argue that the key consideration for this paper is the certainty (or lack thereof) with which findings related to these trials can be extended to people living with frailty. We have added the following section to the discussion expanding on these consideration with specific reference to the GRADE framework and principles. However (as the reviewer points out) there is no specific guidance for GRADE in IPD meta-analyses nor in the assessment of difference in treatment effect, hence we have not included an explicit GRADE assessment.

“Previous meta-analyses of treatments for type 2 diabetes have demonstrated that SGLT2 inhibitors and GLP1 receptor analogues reduce the risk of all-cause mortality, major adverse cardiovascular events, and of end-stage kidney disease with high certainty of evidence according to the grading of recommendations, assessment, development and evaluation (GRADE) framework. We did not apply GRADE to this analysis as our aim was not to assess the overall efficacy of these agents (which is well established, and for which our estimate would be very uncertain due to the exclusion of the large, high quality trials that have established cardiovascular benefits). Our analysis highlights an important limitation of this evidence base for people living with frailty, which is that frailty can only be assessed in a small subset of trials. Cardiovascular outcome trials typically recruit ‘higher risk’ populations based on cardiovascular disease or risk factors. It is currently not clear if these higher-risk trial populations also have a greater degree of frailty. Our findings that trials focusing on older populations or people with chronic kidney disease had higher degrees of frailty but excluded those with the most severe degrees of frailty highlights that the adequate representation of people with frailty cannot be assumed even when inclusion criteria target individuals with a greater health burden.”

Reviewer #3:
Wightman et al., leveraging individual participant data from over 30 randomized trials, examine glucose-lowering therapies and the relevance of frailty (defined via deficit accumulation) in adults with type 2 diabetes. In general, this is an impressive study that tries to fill some of the current data gap underlying clinical guidelines (i.e. recommending higher glycemic targets in adults with frailty, multimorbidity, or otherwise limited life expectancy).

The major point of this work is that the full spectrum of frailty is generally not well-represented in type 2 diabetes trials of newer agents. While in some sense I think this is a result that everyone knows, or at least suspects, the value is really in showing a precise quantification of just how limited the inclusion of frail older adults has been in these trials. I, for one, expected the frailty prevalence estimate to be low, but not generally well below 10%. It is also then not surprising that there was little evidence of treatment effect heterogeneity in these trials, given the more limited range of deficit accumulation scores that tended to be represented.

The major limitation of the work is acknowledged by the authors. They were unable to include the cardiovascular outcome trials due to data limitations in constructing a valid frailty index, which limits what can be said about the benefit side of the equation in terms of cardiovascular prevention. This also makes me wonder how representative the subset of trials examined is of the broader set of trials in this population, given that over 50 of the available trials needed to be excluded.

Thank you for this. We have added a table comparing the included trials with the wider set of eligible trials (with and without IPD). We have also added the following to the results:

“The trials for which we could assess frailty were similar to the wider body of eligible trials in terms of mean age, sex distribution, treatments assessed and type of comparison (detailed in the supplementary appendix). However, while we identified 23 trials that were designed and powered to assess cardiovascular outcomes, and obtained IPD for 6 of these, none of these larger trials had sufficient data on function for us to assess frailty.”

One limitation that the authors should discuss is the focus on treatment effect heterogeneity on the relative scale for MACE and adverse events. This is certainly the typical approach and is also more statistically tractable from a meta-analysis perspective. However, heterogeneity on the absolute scale is likely the more relevant metric for clinical decision making (i.e. a 20% reduction in frail patients is likely a much larger absolute difference versus a 20% reduction in robust patients with lower event rates). The authors could cite the PATH statements in Annals of Internal Medicine, which have a relative concise discussion of this issue.

Thank you. Our presentation of the frailty treatment interactions on the relative scale is consistent with the PATH statement, as our analysis was essentially effect modelling (rather than risk modelling). We agree with the importance of considering absolute risk, particularly in the context of clinical decision making, and with the reviewer’s comment that in the context of MACE and adverse events this is a key consideration. We have expanded the following section of the discussion (new text in bold) along with a reference to the PATH statement.

“We found that a higher frailty index was associated with a range of adverse health outcomes including hypoglycaemia and serious adverse events. **This has important implications when considering the absolute risks and benefits of treatment. While we did not find any difference in the relative effect of treatments on adverse events by frailty status, where the baseline risk of adverse events is higher (as we found to be the case with frailty) then the absolute risk of any treatment associated with adverse events will be greater in these people. Frailty adds the additional dimension of greater vulnerability to decompensation, and it is possible that people living with more severe frailty may experience more severe consequences of adverse events. In this context, the exclusion of those with the most severe frailty from trials makes judging the absolute risks of treatment in this population challenging. It is also important to consider the potential benefits of treatment in absolute terms, and with respect to outcomes that are meaningful to patients.** HbA1c is a surrogate marker which may precede clinical outcomes (of greater relevance to people with diabetes) by many years and may therefore a lesser priority for some older people living with frailty. People living with frailty also had a higher risk of MACE. It is therefore possible that absolute benefits of treatment on MACE may be greater in people with higher frailty index. However, the net-benefit of treatment also depends on the balance between efficacy, adverse events, likelihood of treatment discontinuation, **and competing risks such as non-cardiovascular death.”**

Minor Comments:

1. Line 41. Recognizing that most thresholds for frailty indices are in some sense ad-hoc, the choice of >0.24 for frailty seems a bit weird. I've seen >0.20, >0.21, or even >0.25 used, but never >0.24. Was this a data-driven choice or based on some other justification?

We agree with the reviewer about the lack of consistency with cut-points within the literature. Any cut-point applied is essentially arbitrary (particularly as the frailty index expresses a spectrum of degree of frailty rather than identifying a binary state).

The number 0.24 was based on the threshold for moderate frailty based on the electronic frailty index used in UK primary care. However, we do not seek to draw equivalence between this tool and the application of the frailty index to trial data.

We have amended our presentation of the results to present the proportion of participants across a range of thresholds (0.1, 0.2, 0.3, 0.4) according to recent guidance on frailty index construction (<https://doi.org/10.1093/ageing/afad221>).

We have added the following text to the abstract:

“Using a cut-off of FI>0·2 to indicate frailty, median prevalence was 5.6% (IQR 2.1% to 15.2%). Applying a higher threshold of FI>0.3, median prevalence was 0.3% (IQR 0.1% to 1.2%).”

Methods:

“Based on recent guidance for constructing a frailty index, we did not define ‘frailty’ based on any specific cut-off of the frailty index. Rather, we calculated the proportion of participants in each trial with frailty index values above a range of different thresholds (0.1, 0.2, 0.3 and 0.4). We also provide parameters for the best fitting distributions of each frailty index, allowing re-calculation of the proportion of participants above any given threshold of the frailty index.”

And results…

“The proportion of trial participants for whom the frailty index was >0.2 ranged from 0.6% to 81.9% (median 5.6%, interquartile range 2.1% to 15.2%). Applying a higher cut-off of >0.3, the range was 0.004% to 25.8% (median 0.3%, interquartile range 0.06% to 1.2%). For 33/34 trials, less than 1% of trial participants had a frailty index >0.4 (supplementary appendix)…

The mean age in these trials ranged from 53 to 75 years. However, even in trial participants aged >65 years, the prevalence of frailty was typically low (median 10.4% with frailty index >0·2, interquartile range 4.0% to 621.7%; median 0.5% with a frailty index >0.3, interquartile range 0.08% to 1.9%); supplementary appendix). In trials focused on older people (>70 years, 2 trials) or people with chronic renal impairment (3 trials, one of which focused on severe renal impairment) frailty prevalence was more variable with some trials showing a greater degree of frailty among trial participants (9.0% to 81.9% with frailty index values above 0·2, 0.5 to 25.8% with frailty index values above 0.3).”

2. Line 50-51. I find the results around MACE adverse events confusing. How is this different from the trial MACE outcomes for which the author's report a limited number of events and a high degree of uncertainty? The abstract reads as if the authors have not much to say about MACE, but then somehow there are more statistically certain results?

Thank you for highlighting this. The section of the abstract that the reviewer mentions is referring to the high degree of uncertainty over the effect of treatment on MACE, and any frailty-treatment interaction in this effect. This was limited as there were only 12 trials with over 10 events, and these trials were not powered to assess MACE.

We did, however, assess the association between the baseline frailty index and the incidence of MACE events overall within this 12 trials. We have added some additional text to the abstract to clarify this (added text in bold below):

“Findings for **the effect of treatment on** MACE **(and whether this varied by frailty)** had high uncertainty, with few events occurring in trial follow-up. A 0·1-point increase in the FI was associated with more all-cause adverse events (incidence rate ratio, IRR 1·43, 95% confidence interval 1·34 to 1·53), adverse events judged to the possibly or probably related to treatment (1·35, 1·22 to 1·50), serious adverse events (2·04, 1·80 to 2·30), hypoglycaemia (1·18, 1·04 to 1·34), **baseline risk of** MACE (hazard ratio 3·02, 2·49 to 3·68) and with withdrawal from the trial (odds ratio 1·45, 1·30 to 1·62).”

3. Line 118-119 and 386-387. While the deficit accumulation approach is mostly robust to the precise composition of included deficits, provided they span appropriate domains with sufficient density, there has been some work showing that the impact of variable deficit composition is generally modest, though still not inconsequential (see Shi et al., PMID 32274807). I worry about it a little more in this context given the wide heterogeneity with respect to the functional domain, though I'd agree with the authors that I don't this would strongly impact the study's results.

Thank you for this point and highlighting the Shi et al reference. We have added the following to the discussion section:

“The included trials also lacked any measures in some domains, such as cognition. While the ability of the frailty index to predict adverse outcomes is robust to the selection of deficits provided they cover a range of domains and fulfil the required criteria, omitting deficits from a domain entirely may have a greater impact on the frailty index.”

4. Lines 143-144. I'm curious about the choice to censor at the point of treatment discontinuation. That seems inconsistent with an intention-to-treat approach. Certainly other estimands are justifiable, the authors should just be explicit in terms of what they are estimating, along the lines of the definitions in work such as Kahan et al. (PMID 36790803).

Thank you for highlighting this. In fact the original description in our methods was not entirely accurate. We censored at the point end of available follow-up (rather than treatment discontinuation). We have corrected this in the text, which now reads:

“For our primary analysis, in individuals who were lost to follow-up prior to the primary endpoint the last recorded HbA1c value was carried forward.”

“For each of these outcomes, total follow-up time was also recorded based on the trial baseline, endpoint, and last available followup for participants who withdrew before the primary endpoint.”

5. Lines 161-163. Do the authors have a citation for this statement? While I think this is generally true, it does depend on the model somewhat. For some models, it's more of an approximately correct statement, and I can definitely come up with model structures (i.e. any sort of shared covariance across trials) where this result would not hold.

We agree with the reviewer that one- and two-stage approaches can give different results in certain scenarios. However, as noted by Burke et al. (2016, doi: 10.1002/sim.7141), Riley et al. (2023, doi: 10.1002/jrsm.1661) and others, these differences are typically due to different modelling assumptions between the two approaches (a shared covariance structure across trials mentioned by the reviewer is one example of this, but is not used in this analysis). In this case, we therefore expect the results of one- and two-stage analysis to be equivalent.

Nevertheless, in revision we have removed this statement about the equivalence of the two approaches, since a full discussion is out of scope here, and have instead included a reference supporting the use and equivalence of two-stage IPD MA.

6. Line 171-177. Did the analyses of HbA1c outcome for length of follow-up at all? I see that in the later analyses adverse events, but doesn't seem to be accounted for with HbA1c?

We did not make any adjustment for length of follow-up for the HbA1c results (which was between 6 and 12 months in all included studies), rather analysing efficacy at the point of the primary outcome of each trial. We did, however, examine change in HbA1c over time in response to reviewer 2, finding that our estimates of frailty x treatment interactions were consistent across both of these approaches.

7. Lines 202-209. Can the authors provide any other high-level descriptions that might speak to how the excluded trials might be different from the included trials? Obviously there is the situation of the cardiovascular longer-term outcome trials, but are there any other relevant differences? Perhaps differences along the lines of age, sex, race/ethnicity?

Thank you for this suggestion. We have added a supplementary table comparing the characteristics of all eligible trials, those for which we had IPD, and those for which we could additionally identify sufficient data on health deficits to construct a frailty index. We have added the following to the text highlighting this supplementary table:

“The trials for which we could assess frailty were similar to the wider body of eligible trials in terms of mean age, sex distribution, treatments assessed and type of comparison (detailed in the supplementary appendix).”

8. Lines 398-399. While I think the data here is supportive of this statement, in some sense, the guideline recommendation is a bit of a different animal. Most of these trials reflect the situation of initiating treatment, which is not the same as treatment changes or deprescribing after continued long-term use. There is a distinction there that needs to be made clear.

We have amended to the following:

“Decisions of whether to initiate treatment in people living with frailty should carefully reflect individual goals and priorities, consider guideline recommendations to relax HbA1c-based treatment targets for people living with frailty, and be cognisant of the limited randomised evidence for the balance of risks and benefits in people living with frailty.”

Reviewer #4:
Overall, a well designed and constructed topical study. Frailty and Diabetes have a reciprocal relationship and this make the study even more topical. Important subject at a time where we are finding greater absolute benefits in frail older adults with diabetes given SGLT2i and GLP- 1 RAs i terms of reducing MACE.

I cannot fault the written account of the way in which the literature searching was undertaken to identify the selected studies. The diagrams and figure are excellent. However, my concerns are two fold:

1. These were retrospective studies (with all the accompanying shortfalls of such) in which frailty was clearly not measured and was not part of the original design or research question: constructing the FI from deficits that are available in the trials information is, in my view, subject to bias and potentially inaccurate. In many ways, I view this as cutting corners in frailty detection and identification.

The application of the cumulative deficit model of frailty to secondary data is a well-established practice. Our approach was based on the standard approach for frailty index construction and the included deficits based on established criteria. The quality of such an index then relies on the included deficits themselves and the range of available deficits. We have sought to be as rigorous as possible about our included deficits as well as transparent about their range and limitations.

We ensured that each of the included deficits met the criteria for inclusion in a frailty index, and that each trial included a broad range of deficits (excluding trials that only assessed cardiovascular comorbidities, or that did not assess functional status across a range of measures).

We agree it would likely be ‘better’ if trials explicitly and prospectively assessed frailty using instruments designed for that purpose. However, given that (as the reviewer points out) these trials did not assess frailty as part of the original study design, we are left with the options of applying this retrospective approach as transparently as possible, or concluding that we can make no assessment of frailty in these trials. As these trials have collected rich data across a wide range of domains (which is the basis for the cumulative deficit model of frailty) we would argue that there are important insights to be gained by applying this measure of frailty to these trials, while being open about the limitations of this approach.

We acknowledge the reviewer’s concern that there are drawbacks to this approach when relying on data not collected for the purposes of frailty estimation (or, more broadly, for multidimensional assessment of health across multiple domains). Our approach has been:

1. To be transparent about the range of deficits a trial must include before we attempted to construct a frailty index (i.e. to include both cardiovascular and non-cardiovascular comorbidities, laboratory deficits, and assessment of physical function). We pre-specified these decisions prior to analysing the trial data.
2. Being explicit about what deficits are included in which trial (e.g. Figure 2). This also gives transparency about the variable assessment of functional status across the trials.

The main limitations in our application (which are consistent with the reviewer’s point) are that trials did not assess cognitive function, and that the assessment of physical function was variable across the included studies (and more detailed in some than others). We have expanded the section of the discussion in which we highlight these limitations as below:

“For this to happen, there is a need for trials to adopt inclusion and exclusion criteria that enable people living with frailty to take part; to measure a broad range of comorbidities and functional status to allow frailty to be reliably **assessed (and ideally including additional domains, such as cognition, that were not assessed in these trials)**;[28] to be designed to make it easy for older people living with frailty to participate **(including allowing sufficient time for detailed baseline assessments and adopting approaches to outcome assessment that minimise the burden on participants)**; and to measure clinical endpoints that are relevant to people living with frailty rather than solely assessing surrogate markers.[29]”

2. Frailty detection even using the FI was low in this study but I am not surprised considering the very restricted age range of subjects in this study - you need to go beyond 70 years to increase the likelihood of identifying frailty and complications such as chronic renal disease which increase substantially the risk of frailty.

Our analysis included three trials that focused exclusively on people with chronic kidney disease, and two trials that only recruited people over the age of 70 years (with no upper age limit). Our findings that frailty was greater in these trials, but still well below the levels seen in general population studies is an important observation about these trials which seek to inform the management of these ‘higher risk’ populations.

While the general observation that frailty was uncommon in most trials may not be surprising, we would argue (a) that this is not self-evident without directly examining frailty in these trials and (b) that the representation of frailty is not simply a function of age restrictions or exclusions of comorbidities such as chronic kidney disease (as people with the highest degrees of frailty were under-represented even in trials focused on these populations).

Our findings demonstrate that simply including older people, or those with (e.g.) chronic kidney disease, does not guarantee the inclusion of frailty. This is an important point to demonstrate, as it has implications for both trial design and for the interpretation of existing trial evidence.

Finally, while severe frailty was indeed low, the trials included in our analysis did include people with frailty index values ranging from very low to ‘moderate’ degrees of frailty with clearly demonstrated associations with a range of adverse health outcomes. As such, it shows that the frailty index approach can be used to risk stratify within the trial population, but that caution is required when then applying this to people with greater degrees of frailty than are included within the trials.

My overall impression is that this could have been a very important study for clinical guidelines development if the participants had been mainly in their 70s and 80s, and that accepted frailty measures had been recorded by the individual study investigators at the start of their respective studies. However, I accept that finding such studies may be difficult at the present time. Unfortunately, it means that the findings in this study do not take us forward.

We agree that were people aged over 80 better represented within randomised controlled trials, and if frailty was assessed prospectively using measures selected for this explicit purpose, that meta-analysing such trials would provide better estimates for the use of these treatments that could be incorporated in clinical guidelines. Indeed one of our conclusions is to call for such trials to be conducted (see expanded discussion section below). However, given that trials rarely explicitly measure frailty and older people are under-represented, we are left with the considerable challenge of judging how applicable the evidence base is for people living with frailty. Faced with very limited reporting in of frailty in published trials, we would argue that are findings have value in being explicit about the degree of frailty included within these trials (including, for example, trials focusing specifically on older people which aim to inform the management of these patients).

Put another way, while trials with a low prevalence of frailty are limited in their capacity to inform the management of diabetes in people living with frailty, demonstrating that trials (including, for example, those focusing on people aged over 70) have low levels of frailty demonstrates the importance of caution when applying this evidence base to people living with frailty. Retrospective assessment of frailty (as in this study), allows such judgements about these trials to be based on direct data.

Reviewer #5:
The authors reported on a meta-analysis of RCTs of interactions between frailty index (deficit accumulation model) and antidiabetic drugs such as SGLT2 inhibitors and GLP-1 receptor agonists for HbA1c improvement, MACE, adverse events, and drop-out. The study design and results are very interesting, but concerns have been raised regarding the selection and assessment of the frailty index and its components.

1. In the original study by Rockwood et al, the Frailty Index included chronic conditions, functional status (IADL and ADL), and geriatric syndromes, but not laboratory findings. Because the Frailty Index included laboratory findings, would it have been more likely to produce adverse events? Would similar results be obtained using a frailty index that excluded laboratory findings?

The frailty index approach, be design, can consider different deficits where these are available within different datasets. The inclusion of laboratory deficits within a frailty index is widely carried out and consistent with both the original ‘standardisation’ of the approach to frailty index construction by Searle et al in 2008:

“The principle is to count *deficits in health* (which can be symptoms, signs, diseases, disabilities or laboratory, radiographic or electrocardiographic abnormalities) on the grounds that the more deficits a person has, the more likely that person is to be frail.” (from Searle, S.D., Mitnitski, A., Gahbauer, E.A. Gill, T and Rockwood K*.* A standard procedure for creating a frailty index. *BMC Geriatr* **8**, 24 (2008). <https://doi.org/10.1186/1471-2318-8-24>)

And more recently in updated guidance for frailty index construction:

“A frailty index can be calculated retrospectively from data collected via survey, interview, performance test, laboratory report, clinical or administrative medical record, or any combination of these.” (from Olga Theou, Clove Haviva, Lindsay Wallace, Samuel D Searle, Kenneth Rockwood, How to construct a frailty index from an existing dataset in 10 steps, *Age and Ageing*, Volume 52, Issue 12, December 2023, afad221, <https://doi.org/10.1093/ageing/afad221>)

2. The study defined frailty as a Frailty Index of 0.24 or higher. The cut-off appears to be too high, which would have reduced the prevalence of frailty?

The frailty index represents the spectrum of degrees of frailty and as such any cut-point is essentially arbitrary. This is why we had referred to this as an ‘illustrative cut point’. As reviewer 3 points out, previous studies have used a range of different cut points (with none being considered as an accepted threshold for frailty). We used 0.24 as this is the cut-point termed as ‘moderate frailty’ in the electronic frailty index used in UK primary care – however we also recognized that this is a different operationalization of the frailty index and is not directly transferrable.

We have revised our presentation of results by presenting (in the supplementary appendix) the proportion of trial participants with frailty index values across a range of thresholds (0.1, 0.2, 0.3, 0.4) as recommended in the recently updated guidance for frailty index construction. We would argue this most clearly communicates the core finding of this paper: that there are a range of frailty index values in the trial populations, with some trials having some ‘moderate’ frailty, but that the highest degrees of frailty are rare in trial populations. We have also edited the presentation of the results in the abstract and main text as below:

Methods: “Based on recent guidance for constructing a frailty index, we did not define ‘frailty’ based on any specific cut-off of the frailty index. Rather, we calculated the proportion of participants in each trial with frailty index values above a range of different thresholds (0.1, 0.2, 0.3 and 0.4). We also provide parameters for the best fitting distributions of each frailty index, allowing re-calculation of the proportion of participants above any given threshold of the frailty index.”

Results: “The proportion of trial participants for whom the frailty index was >0.2 ranged from 0.6% to 81.9% (median 5.6%, interquartile range 2.1% to 15.2%). Applying a higher cut-off of >0.3, the range was 0.004% to 25.8% (median 0.3%, interquartile range 0.06% to 1.2%). For 33/34 trials, less than 1% of trial participants had a frailty index >0.4 (supplementary appendix)…

The mean age in these trials ranged from 53 to 75 years. However, even in trial participants aged >65 years, the prevalence of frailty was typically low (median 10.4% with frailty index >0·2, interquartile range 4.0% to 621.7%; median 0.5% with a frailty index >0.3, interquartile range 0.08% to 1.9%); supplementary appendix). In trials focused on older people (>70 years, 2 trials) or people with chronic renal impairment (3 trials, one of which focused on severe renal impairment) frailty prevalence was more variable with some trials showing a greater degree of frailty among trial participants (9.0% to 81.9% with frailty index values above 0·2, 0.5 to 25.8% with frailty index values above 0.3).”

3. Did the ethics of the study population influence the results?

We wonder if this was a typo referring to ethnicity? If so, we did not examine this due to small numbers and incomplete data.
