# Supplementary material for: Frailty in randomized controlled trials of glucose-lowering therapies for type 2 diabetes: An individual participant data meta-analysis of frailty prevalence, treatment efficacy, and adverse events
Source: PLoS Med. 2025 Apr 7;22(4):e1004553. doi: 10.1371/journal.pmed.1004553 (PMC12052138; doi:10.1371/journal.pmed.1004553)
Supplement: Attachment — Submitted filename: Response to editor.docx [file pmed.1004553.s016.docx]

31th January 2025

Dear Dr Van Epps

**Re: Frailty in randomised controlled trials of glucose lowering therapies for type 2 diabetes: An individual participant data meta-analysis of frailty prevalence, treatment efficacy, and adverse events.**

Thank you for assessing our revised manuscript positively and inviting a minor revision adding a comment on limitations to the abstract. We have added the following text:

“The main limitation was that the large cardiovascular outcome trials did not include data on functional status and so we were unable to assess frailty in these larger trials.”

Thank you again, on behalf of the study team, for assessing our manuscript.

Yours sincerely

Dr Peter Hanlon

Clinical Senior Research Fellow

University of Glasgow
